# A Rare Case of Hypomyelinating Leukodystrophy and Its Management: A Case Report and Literature Review

**DOI:** 10.7759/cureus.36471

**Published:** 2023-03-21

**Authors:** Sonali Singh, Anshika Mishra, Chinmayee Murthy, Pugazhendi Inban, Munira Abdefatah Ali, Anupam S Yadav, Tarsha A Intsiful, Zainab T O. Omar, Sakshi Lakhra, Dr.Aadil Khan

**Affiliations:** 1 Pediatrics, King George's Medical University, Lucknow, IND; 2 Internal Medicine, California Institute of Behavioral Neurosciences and Psychology, Fairfield, USA; 3 Department of General Medicine, Government Medical College, Omandurar, Chennai, IND; 4 College of Medicine, University of Illinois at Chicago, Chicago, USA; 5 Psychiatry and Behavioral Sciences, Ganesh Shankar Vidyarthi Memorial (GSVM) Medical College, Kanpur, IND; 6 College of Medicine, University of Ghana Medical Center, Accra, GHA; 7 Pediatrics, Dubai Medical College for Girls, Dubai, ARE; 8 Internal Medicine, All Saints School of Medicine, St. Roseau, DMA; 9 Department of Internal Medicine, Lala Lajpat Rai (LLR) Hospital, Kanpur, IND

**Keywords:** transplantation, rna polymerase, pelizaeus-merzbacher disease (pmd), seizures, hypomyelinating leukodystrophies

## Abstract

A subset of hereditary white matter disorders called hypomyelinating leukodystrophies (HLD) is characterized primarily by the absence of myelin deposition. Although the clinical presentation can be mild and the development of symptoms can occur in adolescence or adulthood, the majority of severe cases present during infancy and early childhood with significant neurological impairments. The clinical features vary from muscle stiffness to seizures and developmental delay. The detailed myelination process can be seen with magnetic resonance imaging (MRI), and many patients are diagnosed using MRI pattern recognition and next-generation sequencing (NGS) in most cases. Here, we report a case of an infant suffering from the hypomyelinating leukodystrophy-13 (HLD-13) variant, whose next-generation sequencing revealed a pathogenic homozygous variant.

## Introduction

Among the inherited white matter disorders, hypomyelinating leukodystrophies (HLDs) are recognized for defective myelin development rather than degradation [1]. These diseases have uniquely distinct findings on magnetic resonance imaging (MRI), such as diminished or absent T2 hypointensity, which typically implies myelin's presence, often without significantly reducing T1 hyperintensity [2]. HLDs consist of bewildering varieties that can occur at any age, of which hypomyelinating leukodystrophy-13 (HLD-13) is rarely reported. Most patients with leukodystrophies have no abnormal physical signs. The clinical presentation is often subtle, and the symptoms usually slowly progress with some possible intervals of stagnation. Vague and unexplained developing motor symptoms or delayed mental development in infancy may be the first clinical manifestation of leukodystrophy. Patients may present with an unusual combination of spasticity and decreased stretch reflexes, or early and recurrent seizure episodes, demonstrating a distinctive aspect of disorders predominantly affecting brain white matter [3]. Here, we report a case of an infant suffering from the HLD-13 variant, whose next-generation sequencing (NGS) revealed a pathogenic homozygous variant, and both parents were found to be carriers of the same genotype [4].

## Case presentation

A seven-month-old baby was brought to the pediatric emergency room with severe bouts of cough and lethargy for the last 24 hours, associated with multiple episodes of vomiting during the previous six days. She had a history of multiple hospitalizations for the last four months due to cough, respiratory distress, hypotonia, and feeding difficulties, requiring mechanical ventilatory support.

She was being managed at a tertiary care hospital as a case of suspected disorders of inborn errors of metabolism with severe sepsis. As per her antenatal history, she was preterm and delivered through a lower-segment Cesarean section with a birth weight of 2 kg. Her five-minute appearance, pulse, grimace, activity, and respiration (APGAR) score was 9, with a head circumference of 29 cm. She had axial hypotonia and limb hypertonia and had three seizure episodes on day 8 of life, for which she received neonatal intensive care. She was managed with anti-epileptics and had no history of any seizure episodes in the next six months. At six months, she again had two episodes of generalized tonic-clonic seizures, for which the dosage of phenobarbital was increased.

**Figure 1:**
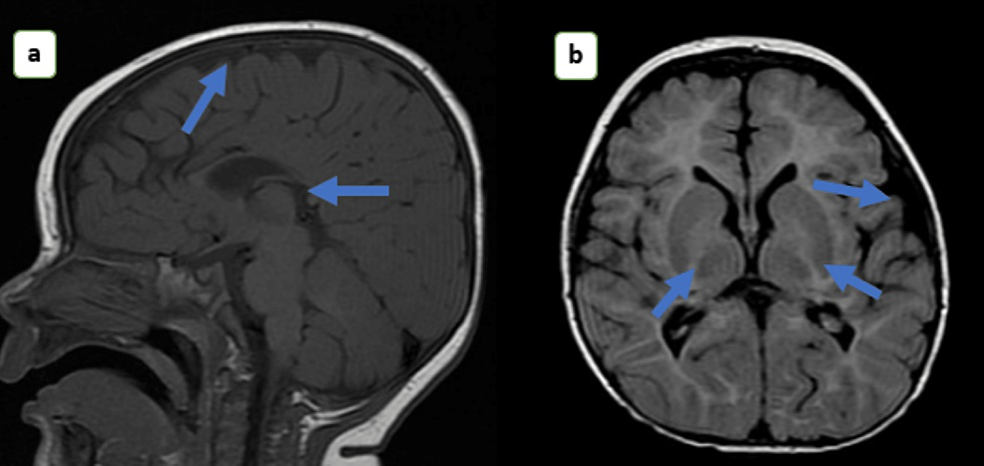
MRI brain showing decreased white matter volume in the bilateral parietal region, right occipital region, diffuse cerebral atrophy, diffuse thinning of the corpus callosum, and minimal myelination of internal capsule, periventricular white matter suggestive leukodystrophy (a, b).

On examination, she was sick and febrile, with a Glasgow coma scale of 5. Her blood pressure and peripheral pulses were not palpable, suggesting hypovolemic shock. Systemic examination revealed global developmental delay and spasticity with brisk deep tendon reflexes. A provisional diagnosis of inborn error of metabolism with septic shock was made. The child was transferred to the pediatric intensive care unit and stabilized with fluid resuscitation, inotropes, ventilatory support, and broad-spectrum antibiotics. She became hemodynamically stable on day 4. Brain MRI showed decreased white matter volume in the bilateral parietal region, right occipital region, and cerebellar hemisphere with prominences of the bilateral lateral and third ventricles, diffuse cerebral atrophy, diffuse thinning of the corpus callosum, minimal myelination of the internal capsule, and periventricular white matter suggestive of leukodystrophy (Figure 1).

Gas chromatography, mass spectrometry, and tandem mass spectrometry were negative. Whole genome sequencing suggested a likely homozygous variant (HIKESHI) consistent with the phenotype detected in HLD-13; the same variant was seen in heterozygous form in her parents. Gradually, her ventilator support was weaned off and discontinued on day 8. Ryles tube (RT) feeding was started and built gradually, and anti-epileptics were upgraded. After one month of follow-up, she was still on the RT feed but was seizure-free with no deterioration.

## Discussion

An extensive and persistent impairment in the central nervous system's (CNS) myelin deposition characterizes the varied group of hereditary white matter illnesses known as HLD [5]. Leukodystrophies can manifest at any age, from premature infants and newborns to adults in their late years. Cases have been reported from all racial groups and geographical areas worldwide. Merzbacher, who studied brain tissue from a patient reported by Friedrich Pelizaeus 25 years earlier, first identified this trait in 1910 [6]. The condition they described quickly became known as Pelizaeus-Merzbacher disease (PMD), which is currently regarded as the model hypomyelinating ailment. Pelizaeus previously suspected that PMD was inherited on the X chromosome. Still, it took more than a century after his initial description of the underlying gene defect - variants in the gene encoding the most abundant myelin protein, proteolipid protein 1 (PLP1) - to be discovered [7]. There are several hypomyelinating leukodystrophies that are more well-known, including PMD, RNA polymerase III-related leukodystrophies/4-H syndrome (hypomyelination, hypogonadotropic hypogonadism, and hypodontia), and hypomyelination with atrophy of the basal ganglia and cerebellum. Even the same person may have an illness that fits into multiple categories. Furthermore, this theme of categorization is mainly based on the ambiguous magnetic resonance imaging (MRI) appearance of leukodystrophy. Motor delay is more common in hypomyelinating leukodystrophies than motor regression, which is more common in demyelinating leukodystrophies. Characteristic brain patterns and the use of quantitative MRI techniques can offer measurements of the myelin content of the white matter, serving as biomarkers for hypomyelinating diseases.

The age at which symptoms or developmental regression first appear may also aid in delineating the differential diagnosis. Although most leukodystrophies have infantile, juvenile, and adult variants, one of these categories is usually more prevalent. Aicardi-Goutieres syndrome and PMD are the most prevalent types, and both have symptoms that appear at birth or within the first few months of life [8]. Most Krabbe disease patients (90%) have symptoms between 6 and 12 months of age [9]. The most prevalent type is the late-infantile variant of metachromatic leukodystrophy (MLD), with symptoms beginning between one and two years of age. Boys with X-linked adrenoleukodystrophy (X-ALD) experience neurological symptoms between the ages of four to ten most frequently. For hypomyelinating diseases, there are currently no causative treatments available, but therapeutic advances like gene therapy may be possible due to the advancement in our understanding of the genetic origins of hypomyelination.

Transplantation of healthy glial progenitor cells to promote myelination is another potential therapy option for illnesses that cause hypomyelination. The cell transplantation of oligodendrocytes showed efficacy in a PMD mouse model. However, it had no beneficial clinical benefits and produced no significant myelination that could be seen on MRI in four PMD patients [10]. Although some centers offer hematopoietic stem cell transplantation, it is not a feasible method since transplanted cells develop into pathogenic macrophagic cells instead of oligodendrocytes [11]. Enhancing axonal metabolic support is a fascinating therapeutic approach that is being tested in animal models. Treatment with the ketogenic diet to obtain this axonal metabolic support boosted myelination and improved axonal survival in PMD mice [12]. As cholesterol cannot cross the blood-brain barrier (BBB), the ketogenic diet can overcome this problem because of its ability to cross the BBB, but a cholesterol-rich diet works similarly in rats. Another risk factor is defective iron metabolism. In PMD, oligodendrocytes were discovered to have aberrant iron metabolism, and the survival and myelination of oligodendrocytes can be increased using iron chelators [13].

## Conclusions

Hypomyelinating leukodystrophies-13 is a rare inherited hypomyelination disorder affecting various age groups. The disease can manifest with vague symptoms ranging from minor stiffness to major intractable seizures and motor delay in infants, and may even cause death. However, this dreaded disease has no specific imaging technique, treatment, or cure. Currently, the only treatments that provide satisfactory improvement in halting disease progression are an increased ketogenic diet and iron-rich food items. Researchers are conducting numerous experiments and trials on animals, and treatments and cures are soon expected.

## References

[1] Pouwels PJ, Vanderver A, Bernard G (2014). Hypomyelinating leukodystrophies: translational research progress and prospects. Ann Neurol.

[2] Ashrafi MR, Amanat M, Garshasbi M (2020). An update on clinical, pathological, diagnostic, and therapeutic perspectives of childhood leukodystrophies. Expert Rev Neurother.

[3] Kohlschütter A, Eichler F (2011). Childhood leukodystrophies: a clinical perspective. Expert Rev Neurother.

[4] Yan H, Ji H, Kubisiak T (2021). Genetic analysis of 20 patients with hypomyelinating leukodystrophy by trio-based whole-exome sequencing. J Hum Genet.

[5] van der Knaap MS, Schiffmann R, Mochel F, Wolf NI (2019). Diagnosis, prognosis, and treatment of leukodystrophies. Lancet Neurol.

[6] Merzbacher L (1910). Eine eigenartige familiär-hereditäre erkrankungsform (Aplasia axialis extracorticalis congenita). Neur Psych.

[7] Hudson LD, Puckett C, Berndt J, Chan J, Gencic S (1989). Mutation of the proteolipid protein gene PLP in a human X chromosome-linked myelin disorder. Proc Natl Acad Sci U S A.

[8] Hobson GM, Garbern JY (2012). Pelizaeus-Merzbacher disease, Pelizaeus-Merzbacher-like disease 1, and related hypomyelinating disorders. Semin Neurol.

[9] Wasserstein MP, Andriola M, Arnold G (2016). Clinical outcomes of children with abnormal newborn screening results for Krabbe disease in New York State. Genet Med.

[10] Gupta N, Henry RG, Kang SM (2019). Long-term safety, immunologic response, and imaging outcomes following neural stem cell transplantation for Pelizaeus-Merzbacher disease. Stem Cell Reports.

[11] Wolf NI, Breur M, Plug B (2020). Metachromatic leukodystrophy and transplantation: remyelination, no cross-correction. Ann Clin Transl Neurol.

[12] Stumpf SK, Berghoff SA, Trevisiol A (2019). Ketogenic diet ameliorates axonal defects and promotes myelination in Pelizaeus-Merzbacher disease. Acta Neuropathol.

[13] Nobuta H, Yang N, Ng YH (2019). Oligodendrocyte death in Pelizaeus-Merzbacher disease Is rescued by iron chelation. Cell Stem Cell.

